# Genomic Counter-Stress Changes Induced by the Relaxation Response

**DOI:** 10.1371/journal.pone.0002576

**Published:** 2008-07-02

**Authors:** Jeffery A. Dusek, Hasan H. Otu, Ann L. Wohlhueter, Manoj Bhasin, Luiz F. Zerbini, Marie G. Joseph, Herbert Benson, Towia A. Libermann

**Affiliations:** 1 Benson-Henry Institute for Mind Body Medicine at Massachusetts General Hospital, Chestnut Hill, Massachusetts, United States of America; 2 Department of Psychiatry, Massachusetts General Hospital, Harvard Medical School, Boston, Massachusetts, United States of America; 3 Department of Medicine, Division of Interdisciplinary Medicine and Biotechnology, Beth Israel Deaconess Medical Center, Harvard Medical School, Boston, Massachusetts, United States of America; 4 BIDMC Genomics Center, Beth Israel Deaconess Medical Center, Boston, Massachusetts, United States of America; 5 Department of Medicine, Massachusetts General Hospital, Harvard Medical School, Boston, Massachusetts, United States of America; 6 Institute for Health and Healing, Abbott Northwestern Hospital, Minneapolis, Minnesota, United States of America; University of Montreal, Canada

## Abstract

**Background:**

Mind-body practices that elicit the relaxation response (RR) have been used worldwide for millennia to prevent and treat disease. The RR is characterized by decreased oxygen consumption, increased exhaled nitric oxide, and reduced psychological distress. It is believed to be the counterpart of the stress response that exhibits a distinct pattern of physiology and transcriptional profile. We hypothesized that RR elicitation results in characteristic gene expression changes that can be used to measure physiological responses elicited by the RR in an unbiased fashion.

**Methods/Principal Findings:**

We assessed whole blood transcriptional profiles in 19 healthy, long-term practitioners of daily RR practice (group M), 19 healthy controls (group N_1_), and 20 N_1_ individuals who completed 8 weeks of RR training (group N_2_). 2209 genes were differentially expressed in group M relative to group N_1_ (p<0.05) and 1561 genes in group N_2_ compared to group N_1_ (p<0.05). Importantly, 433 (p<10_−10_) of 2209 and 1561 differentially expressed genes were shared among long-term (M) and short-term practitioners (N_2_). Gene ontology and gene set enrichment analyses revealed significant alterations in cellular metabolism, oxidative phosphorylation, generation of reactive oxygen species and response to oxidative stress in long-term and short-term practitioners of daily RR practice that may counteract cellular damage related to chronic psychological stress. A significant number of genes and pathways were confirmed in an independent validation set containing 5 N_1_ controls, 5 N_2_ short-term and 6 M long-term practitioners.

**Conclusions/Significance:**

This study provides the first compelling evidence that the RR elicits specific gene expression changes in short-term and long-term practitioners. Our results suggest consistent and constitutive changes in gene expression resulting from RR may relate to long term physiological effects. Our study may stimulate new investigations into applying transcriptional profiling for accurately measuring RR and stress related responses in multiple disease settings.

## Introduction

The relaxation response (RR) has been defined as a mind-body intervention that offsets the physiological effects caused by stress [1], [2]. The RR has been reported to be useful therapeutically (often as an adjunct to medical treatment) in numerous conditions that are caused or exacerbated by stress [3]–[6].

Mind-body approaches that elicit the RR include: various forms of meditation, repetitive prayer, yoga, tai chi, breathing exercises, progressive muscle relaxation, biofeedback, guided imagery and Qi Gong [7]. One way that the RR can be elicited is when individuals repeat a word, sound, phrase, prayer or focus on their breathing with a disregard of intrusive everyday thoughts [2]. The non-pharmacological benefit of the RR on stress reduction and other physiological as well as pathological parameters has attracted significant interest in recent years to decipher the physiological effects of the RR. In addition to decreased oxygen consumption [8]–[10], other consistent physiologic changes observed in long-term practitioners of RR techniques include decreased carbon dioxide elimination, reduced blood pressure, heart and respiration rate [1], [2], [11], prominent low frequency heart rate oscillations [12] and alterations in cortical and subcortical brain regions [13], [14].

Despite these observations and the well-established clinical effects of RR-eliciting practices [15], [16], the mechanisms underlying the RR have not been identified. Similarly, the impact of the RR on gene expression and signaling pathways has not yet been explored in detail, although a transcriptional profiling study of Qi Gong [17] practitioners, another RR method, revealed apparent distinct gene expression differences between Qi Gong practitioners and age matched controls. It is likely that differences in gene expression may be an underlying factor in the physiologic and psychologic changes noted above. Toward that end, we conducted a study to explore the gene expression profile of healthy long-term practitioners versus healthy age and gender matched controls. As a further evaluation, we provided 8-weeks of RR training to the control subjects and re-assessed their gene expression.

## Results

### Patient characteristics

This study includes both cross sectional and an 8-week prospective design. Healthy adults were enrolled, comprising 2 groups: individuals with a long-term RR practice (group M; n = 19) or those with no prior RR experience (novice; group N_1_; n = 19). Group N_1_ novices, furthermore, underwent 8-weeks of RR training (Group N_2_; n = 20) for the prospective analysis. In the cross sectional study, we compare gene expression profiles (GEP) in whole blood between groups M and N_1_, whereas in the prospective study GEP is compared for each individual novice subject before and after RR experience, matched individuals of groups N_1_ versus N_2_ respectively.

### Gene expression changes associated with the RR

**Figure 1:**
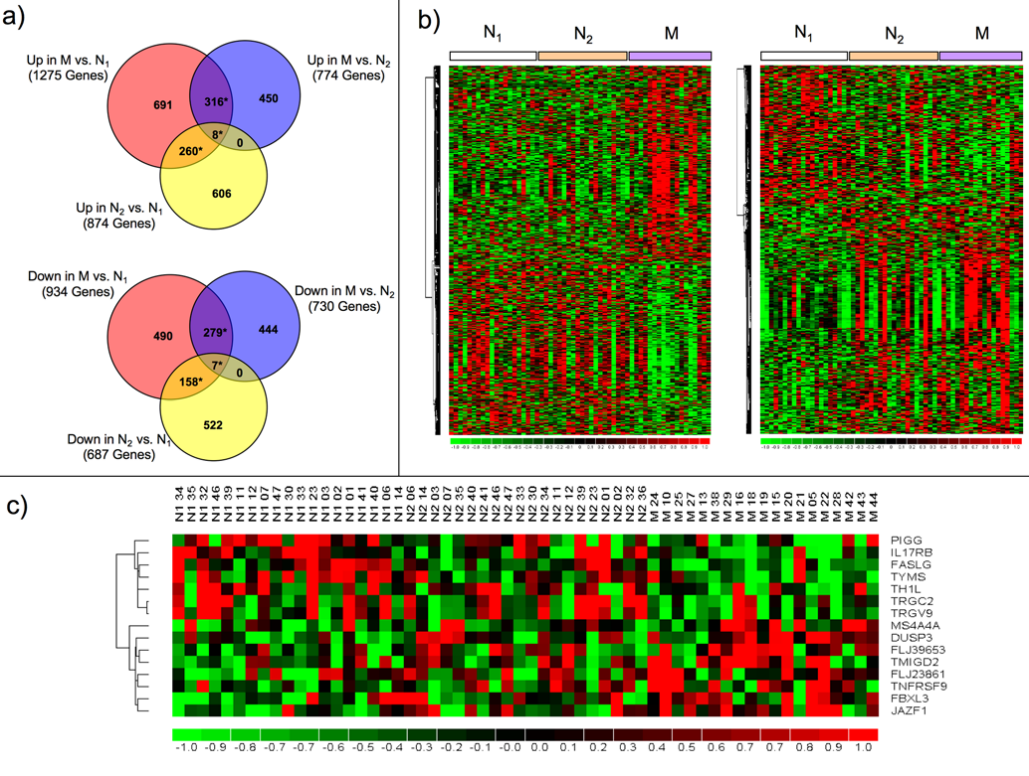
Gene Ontology Analysis. Analysis of differentially expressed genes: a) Venn diagrams: * indicates significant overlaps (p<10^6^); b) Heatmaps of the 595 differentially regulated genes in both M vs. N_1_ and M vs. N_2_ (left) and the 418 differentially regulated genes in both M vs. N_1_ and N_2_ vs. N_1_; c) Heatmap of 15 genes in the intersection of all three groups (gene symbols listed on the right). In heatmaps, rows represent genes and columns represent samples from N_1_, N_2_, and M groups. Genes are clustered using row-normalized signals and mapped to the [−1,1] interval (shown in scales beneath each heatmap). Red and green represent high and low expression values, respectively.

Transcriptional differences between the different groups and within individuals before and after the RR are assessed by microarray analysis using Affymetrix HG-U133 Plus 2.0 genechips (www.affymetrix.com). This technology is a well established and reliable method to assess global gene expression differences [18]. Comparing group M (subjects with long term RR practice) to group N_1_ (subjects prior to RR training), we find statistically significant differential expression of 2209 genes; 1275 significantly up-regulated and 934 significantly down-regulated in M vs. N_1_. Additionally, 1561 genes are differentially expressed in novices after RR experience, N_2_ vs. N_1_; 874 significantly up-regulated and 687 significantly down-regulated. Comparison of gene lists from M vs. N_1_, N_2_ vs. N_1_ and M vs. N_2_ with Venn diagrams reveals significant overlap (Fig. 1a). Significance of overlaps is calculated using hypergeometric distributional assumption [19] and p-values are adjusted using Bonferroni correction for multiple comparisons [20]. Heatmaps were generated from genes in the intersecting areas of the Venn diagrams (Fig. 1b). We find 316 up-regulated and 279 down-regulated genes are differentially expressed in group M compared to both group N_1_ and N_2_; these changes in GEP are only observed in long-term RR practitioners. Similarly, 260 genes are up-regulated and 168 genes are down-regulated in both groups M and N_2_ compared to N_1_; they represent GEP changes characteristic of RR practice over at least 8 weeks.

Heatmaps generated using these genes exhibit consistent GEP changes across the three groups with a few samples in each group resembling the GEP of another group. To determine if any demographic characteristics (e.g. age, ethnicity , etc.) influences this observation, we clustered each group separately using the same set of genes. For each cluster analysis, we calculated the significance of observing a characteristic among the samples in the subgroups formed. We found that number of times M subjects reported eliciting the RR per week was significantly associated with the subgroups formed when M samples were clustered using genes differentially expressed in long term RR practitioners only. Specifically, there were 316 up-regulated and 279 down-regulated genes differentially expressed in group M compared to both group N_1_ and N_2_; (Fig. 1b). All remaining cluster analyses revealed no such significant influence of demographic characteristics (see online supplementary data).

Finally, the intersection of all 3 areas (M vs. N_1_, N_2_ vs. N_1_ and M vs. N_2_) identifies genes with expression behavior that is monotonically changed between N_1_ to N_2_ to M (Fig. 1c). These results clearly demonstrate that short term as well as long term RR practice lead to distinct and consistent gene expression changes in hematopoietic cells.

### Signaling pathways modulated by the RR

We performed Expression Analysis Systematic Explorer (EASE) analysis [21] using M vs. N_1_, and N_2_ vs. N_1_ data-sets, to identify Gene Ontology (GO) categories where specific genes in these data occur more often than would be expected by random distribution of genes. These findings (and those of the validation data-set below) are summarized in Table 1, where select over-represented GO categories are listed along with specific genes differentially expressed in our data-sets. These categories include oxidative phosphorylation, ubiquitin-dependent protein catabolism, nuclear messenger RNA (mRNA) splicing, ribosomes, metabolic processes, regulation of apoptosis, NF-κB pathways, cysteine-type endo-peptidase activity and antigen processing. Most are significant in both long-term (M vs. N_1_) and short-term (N_2_ vs. N_1_) practitioners of daily RR practice (see Table).

**Table 1:** Gene Ontology Categories

SELECTED GENE ONTOLOGY CATEGORY	Original Data-set (n = 58)		Validation Data-set (n = 16)	
	M vs. N1 (2209)	N2 vs. N1 (1561)	M vs. N1 (1846)	N2 vs. N1 (2390)
**OXIDATIVE PHOSPHORYLATION (84)**	20†	7	7	20†
	*ATP5E;ATP5L; COX5B, COX7B; NDUFB2; UCRC; UQCRB*	*ATP5E; NDUFB2; UQCRC2*	*ATP5L; NDUFB2; UQCRB*	*ATP5L; COX5B;COX7B; NDUFB2; UCRC; UQCRB;UQCRC2*
**UBIQUITIN-DEPENDENT PROTEIN CATABOLISM (127)**	19*	16*	21**	17
	*ANAPC4; PSMB2; PSMF1; USP14; USP48*	*PSMB2; PSMF1; USP14*	*ANAPC4; PSMB2; PSMF1; USP14; USP48*	*ANAPC4; PSMB1; USP48*
**NUCLEAR MESSENGER RNA SPLICING, VIA SPLICOSOME (127)**	22**	15*	18*	21*
	*HNRPH3; TARDBP; THOC2; U2AF2*	*HNRPH3; TARDBP; THOC2*	*TARDBP; THOC2; U2AF2*	*HNRPH3; THOC2; U2AF2*
**RIBOSOME (171)**	64‡	20*	31‡	33†
	*RPL13A; RPL23A; RPL37A; RPS27*	*RPL13A; RPL23A; RPL37A; RPS27*	*RPL13A; RPL37A; RPS27*	*RPL23A;RPL37A; RPS27*
**PRIMARY METABOLISM (6379)**	667‡	477‡	574†	694†
	*ADCY9; CLC; PCK2; PDHA1; SOD2*	*ADCY9; CLC; KLF6; PCK2; PDHA1*	*ADCY9; CLC*	*KLF6; SOD2*
**NEGATIVE REGULATION OF METABOLISM (238)**	31*	24*	30*	35*
	*HDAC8; MDM4; PPARD; TH1L*	*MDM4; SIRT2; TH1L; ZBTB16*	*ZBTB16*	*TH1L; ZBTB16*
**REGULATION OF APOPTOSIS (343)**	38	37**	48†	42
	*BIRC4; CFLAR; FAS; PRDX2; PRDX5*	*BIRC4; CFLAR; HSP90B1; PRDX2; PRDX3*	*CFLAR*	*CFLAR*
**REGULATION OF I-kB KINASE/NF-Κ B CASCADE (83)**	12	14**	14*	13
	*FASLG; HMOX1; TNFSF10*	*FASLG; IKBKE; TNFSF15; TRAF6*	*HMOX1*	*IKBKE*
**CYSTEINE-TYPE ENDO-PEPTIDASE ACTIVITY (105)**	16	13*	17*	18*
	*ATG4D; CASP2; CASP9*	*CASP2; CASP7; CTSB*	*CASP2; CTSB*	*ATG4D*
**ANTIGEN PROCESSING (34)**	9*	11‡	8*	13‡
	*HFE; HLAC; LRAP*	*HFE; HLAC*	*HFE; HLAC*	*HFE; LRAP*

Numbers in parentheses are the total number of genes per comparison for each data-set or the GO reference set. The number of differentially expressed genes with representative members in that GO category for each comparison and data-set are listed. Significance of EASE scores are indicated as follows: p<0.05^*^, p<0.01^**^, p<0.001^†^, p<0.0001^‡^.

**Figure 2:**
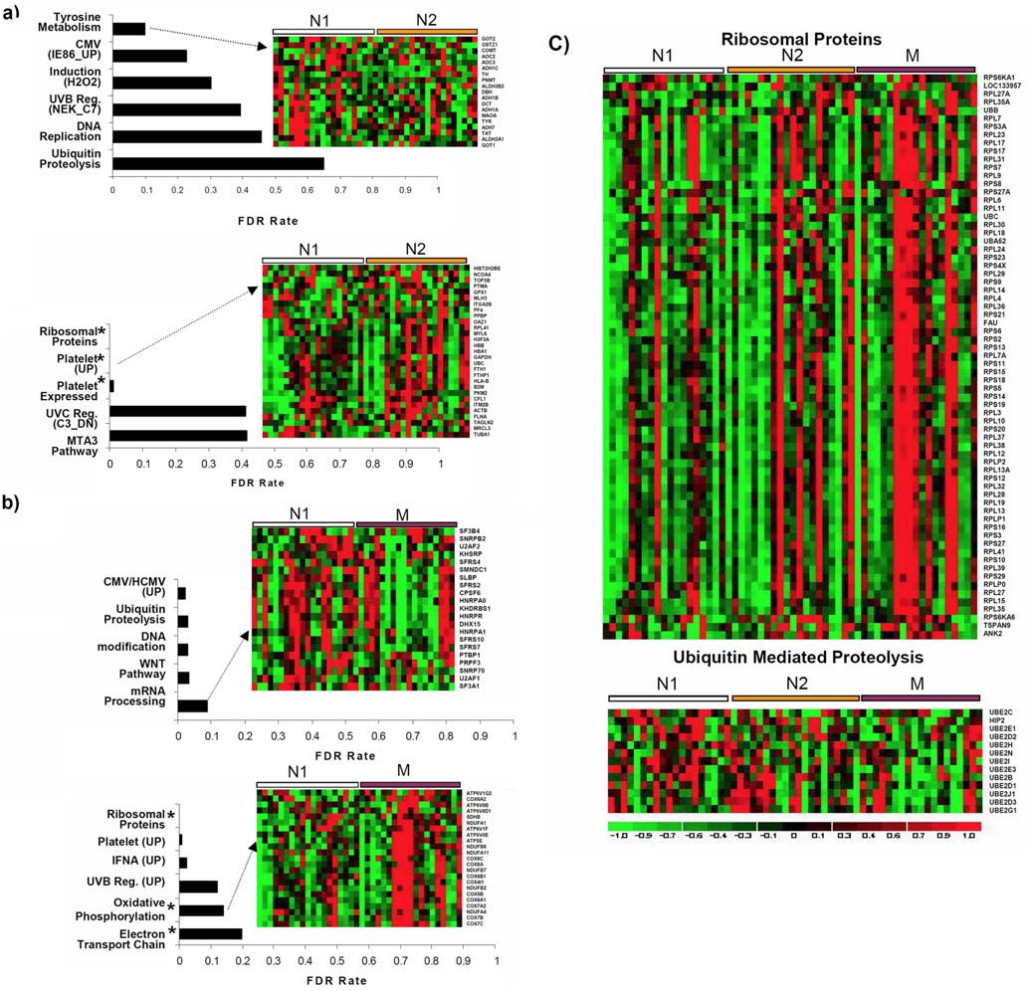
GSEA Analysis. The analysis has been performed for >1200 predefined datasets using GSEA 2.0 software. Signal values for each gene are obtained by collapsing the probe values using max_probe algorithm. Representative datasets, significantly enriched (FDR<50%, or NPV< = 0.01) between any two groups and corresponding heatmaps (depicting relative gene expression changes of core enrichment) are shown in a) N_2_ vs. N_1_ and b) M vs. N_1_. Datasets that are enriched in both the original and validation analyses are marked with *. c) Heatmaps of ribosomal proteins and ubiquitin mediated proteolysis illustrate transitional trends in gene expression across the N_1_, N_2_ and M groups.

Even though our analyses of differentially expressed genes and GO categories associated with RR practice meet widely accepted criteria for statistical significance, we were concerned about the relatively small fold changes that were observed (see Supplementary Methods). To address this issue we employed Gene Set Enrichment Analysis (GSEA). GSEA has proven to be useful for capturing subtle expression changes in complex gene signatures based on predefined gene sets or pathways [22]. As described above, we examined expression data for 2 comparisons, M vs. N_1_ and N_2_ vs. N_1_. The selected pathways or gene sets that are significantly enriched (False Discovery Rate (FDR)<50%, nominal p-value (NPV)< = 0.02) are shown in Figure 2, with gene sets for N_2_ vs. N_1_ and M vs. N_1_ in Fig 2A and Fig 2B respectively.

GSEA analysis of N_2_ vs. N_1_ showed highly significant enrichment in gene sets related to various cellular stressors/stress responses and metabolism. To a pronounced degree these observations complement the results of GO analysis presented in the Table, also depicting significant alterations in cellular response to stress, oxidative and primary metabolism. The transition effect of the RR from novice to short term (8 weeks) to long term RR practitioners has been denoted through a colorgram of ribosomal proteins and ubiquitin mediated proteolysis gene sets (Fig. 2C). Whereas expression of ribosomal genes is significantly upregulated in RR practitioners at 8 weeks and more pronounced in long term practitioners, ubiquitin mediated proteolysis gene expression in general shows an opposite trend. Closer inspection of the colorgrams for ribosomal proteins and Ub proteolysis gene sets shows some variation in the GEP in each subgroup (N_1_, N_2_ M). The GEP of a few N_1_ or N_2_ subjects resembles the GEP of M subjects and vice versa. To elucidate the association between GEP and subject characteristics (Race, Age, etc), we performed clustering of each subgroup separately (N_1_, M) using the enriched gene sets (Ribosomal and Ub Proteolysis). This analysis identified a subcluster in the N_2_ subgroup that has significant over-representation of Asian subjects (P value <0.05) when the clustering was performed using the ribosomal protein gene set. This observation needs further validation on a larger dataset as the current study contains only five Asian subjects. No other characteristic exhibited significant association with the ribosomal protein gene set. No significant association between the GEP profiles and subject characteristic was found when clustering was performed using the Ub proteolysis gene set. This analysis provides further insight into the stress response related genes that are influenced by RR practice.

### Independent validation set analysis

As a validation of our results, we repeated the experimental and analysis procedures defined in the “Methods” section on a new set of samples consisting of 5 N_1_, 5 N_2_ and 6 M subjects. We found 1846 and 2390 probe sets differentially expressed between M vs. N_1_, and N_2_ vs. N_1_ groups. The validation data-set showed a significant (p<10^−5^) number of genes in common with the original analysis of 58 samples. We also found that 70–75% of all GO categories from the original analysis were retained in the validation set (p ∼0), and 30–65% of significantly over-represented GO categories were shared. Of note, biologically relevant GO categories such as oxidative phosphorylation, regulation of apoptosis, and antigen presentation, come up as significantly over-represented in both the original and validation analyses. Results of the validation set and comparison analyses can be found in the online supplementary data. In addition, validation GSEA analysis on N_2_ vs. N_1_ subjects shows enrichment of ribosomal proteins and platelet expressed gene sets and enrichment of ribosomal proteins, oxidative phosphorylation and electron transport chain gene sets in M vs. N_1_ subjects (Fig. 2A and 2B). The similarities between the original and validation results from GSEA analysis argues against random chance accounting for the observed enrichment of these gene sets.

## Discussion

Results from our study indicate that there are distinct differences in the GEPs between individuals with many years of RR practice (group M) and those without such experience (group N_1_). Furthermore we find significant GEP changes within the same individuals before (N_1_) and after 8 weeks of RR training (N_2_). Finally, the changes in GEP found in M vs. N_1_, and those of N_2_ vs. N_1,_ are to a great degree similar when assessed by analysis of differentially expressed genes, GO analysis and GSEA.

It is becoming increasingly clear that psychosocial stress can manifest as system-wide perturbations of cellular processes, generally increasing oxidative stress and promoting a pro-inflammatory milieu [23]–[25]. Stress associated changes in peripheral blood leukocyte expression of single genes have been identified [26]–[28]. More recently, chronic psychosocial stress has been associated with accelerated aging at the cellular level. Specifically, shortened telomeres, low telomerase activity, decreased anti-oxidant capacity and increased oxidative stress are correlated with increased psychosocial stress [29] and with increased vulnerability to a variety of disease states [30]. Stress-related changes in GEP have been demonstrated by microarray analysis in healthy subjects, including up-regulation of several cytokines/chemokines and their receptors [31], and in individuals suffering from post-traumatic stress disorder, including inflammation, apoptosis and stress response [32] as well as metabolism and RNA processing pathways [33]. The pro-inflammatory transcription factor NF-kappa B (NF-κB) which is activated by psychosocial stress has been identified as a potential link between stress and oxidative cellular activation [34].

The RR is clinically effective for ameliorating symptoms in a variety of stress-related disorders including cardiovascular, autoimmune and other inflammatory conditions and pain [15]. We hypothesize that RR elicitation is associated with systemic gene expression changes in molecular and biochemical pathways involved in cellular metabolism, oxidative phosphorylation/generation of reactive oxygen species and response to oxidative stress and that these changes to some degree serve to ameliorate the negative impact of stress. Genome-wide evaluation of PBL GEP is a reasonable approach to survey the transcriptional changes that are involved in elicitation of the RR. The GEP of RR practitioners presented here reveals altered gene expression in specific functional groups which suggest a greater capacity to respond to oxidative stress and the associated cellular damage. Genes including COX7B, UQCRB and CASP2 change in opposite direction from that in the stress response [31], [32].

Our findings are relatively consistent with those found in a study of Qi Gong [17], a practice that elicits the RR. In their study of 6 Qi Gong practitioners and 6 aged matched controls, practitioners had down-regulation of ubiquitin, proteasome, ribosomal protein and stress response genes and mixed up- and down-regulation of genes involved in apoptosis and immune function. We find a similar pattern of GO categories that are significantly over-represented in GO or enriched in GSEA in our cross sectional comparison, M vs. N_1_. However, in our data-set ribosomal proteins were up-regulated.

Overall, similar genomic pattern changes occurred in practitioners of a specific mind body technique (Qi Gong) as well as in our long-term practitioners who utilized different RR practices including Vipassna, mantra, mindfulness or transcendental meditation, breath focus, Kripalu or Kundalini Yoga, and repetitive prayer. This indicates there is a common RR state regardless of the techniques used to elicit it.

Our study is the first to prospectively evaluate GEP changes in individuals before and after a short-term (8 week) RR training which consequently enables an appreciation of the parallel GEP changes that occur with short- and/or long-term RR practice. Replications in larger cohorts are warranted. Future investigations could better define the therapeutic value and required duration of RR training to counter stress-related disorders.

## Materials and Methods

### Participants

Nineteen healthy practitioners of various RR eliciting techniques (including several types of meditation, Yoga, and repetitive prayer) participated (M group; n = 19). Years of practice averaged 9.4 years (5.0 sd) and ranged from 4 to 20 years. Twenty individuals without any prior RR eliciting experience served as controls (N group; n = 20).. As shown in Table 2, the M and N groups are matched with respect to age, gender, race, height, weight, and marital status, which do not exhibit significant difference between the groups (p>0.05, t- and chi-square test).

**Table 2:** Demographics

	N group	M group	p-value
**Age**	36.68±6.22-3	37.21±6.93	0.81
**Race**	**10** White, **4** Asian,	**16** White, **1** Asian,	0.15
	**3** African American,	**2** African American,	
	**2** Hispanic	**0** Hispanic	
**Gender**	**9** Male, **10** Female	**9** Male, **10** Female	1.0
**Height**	66.32±3.73	68.79±4.22	0.06
**Weight**	152.47±24.40	153.58±16.82	0.87
**Marital Status**	**4** Married, **1** Widowed,	**5** Married, **0** Widowed,	0.73
	**3** Seperated/Divorced,	**4** Seperated/Divorced,	
	**11** Never Married	**10** Never Married	

The demographic characteristics for the N and M groups. The age, height, and weight p-values were calculated using t-test, whereas the race, gender, and marital status p-values were calculated using chi-square test. There were no significant differences across the groups

### Protocol

The study protocol was approved by the Committee on Clinical Investigations at the Beth Israel Deaconess Medical Center (BIDMC), Boston MA. All subjects provided written informed consent and the study was conducted in the General Clinical Research Center (GCRC) of the BIDMC. After providing written informed consent, participants were screened by a physician and had blood drawn to ensure good health. All participants completed a testing session in the GCRC. N_1_ (novice) subjects had 8-weeks of RR training, listened to a 20-minute RR-eliciting CD daily and returned to the GCRC for a repeat testing session (hereafter classified as the N_2_ group).

### Relaxation-Response Training

N subjects received 8 weeks of RR training. Training included information about reducing daily stress, and a 20-minute elicitation of the RR [35]. Subjects randomized to the RR group received 8 weekly individual RR-training sessions from an experienced clinician as per our manualized research protocol [35]. The first session provided an educational overview of the stress response and the RR, instructions on how to elicit the RR, and a 20-minute guided RR experience. Sessions 2 through 8 consisted of a review of the subject's home practice card for compliance and a 20-minute guided RR experience.

During the RR elicitation in the weekly session, the subject was guided through a RR sequence including: diaphragmatic breathing, body scan, as well as mantra and mindfulness meditation, while subjects passively ignored intrusive thoughts. The specific CD guided the subject through the same sequence and has our clinical research studies and clinical practice for more than 15 years [35]. Subjects were asked to listen to the RR-eliciting CD once a day for 20 minutes at home.

To measure compliance, participants' daily home practice logs were reviewed each week and at the end of the 8 week training. These logs indicate that N subjects listened for an average of 17.5 minutes per day (3.7 sd) over 8-weeks.

### Microarray Analysis

Following previously described protocols, the transcriptional profile of samples were probed using Affymetrix HG-U 133 Plus 2.0 chips representing over 47,000 transcripts and variants using more than 54,000 probesets. Scanned image output files were visually examined for major chip defects and hybridization artifacts and then analyzed with Affymetrix GeneChip Microarray Analysis Suite 5.0 (MAS5) software (Affymetrix). The image from each chip was scaled such that the 2% trimmed mean intensity value for all arrays was adjusted to target intensity and reported as a non-negative quantity. Chips used for subsequent analysis consisted of 19 M, 19 N_1_ and 20 N_2_ samples (one chip from the N_1_ group had insufficient signal values). A hierarchical clustering technique was used to construct an Unweighted Pair Group Method with Arithmetic-mean (UPGMA) tree using Pearson's correlation as the metric of similarity [36]. The expression data matrix was row-normalized for each gene prior to the application of average linkage clustering. When comparing 2 groups of samples to identify genes enriched in a given group, we used combination of three criteria. We considered genes with significantly different expression across the two groups using t-test (p<0.05) that further remained significant at a 5% false discovery rate (FDR) using permutation testing with 1,000 permutations [37], [38]. In order to finalize a set of genes significantly up-regulated in a given group compared to another group, among the genes that passed the aforementioned steps, we filtered the ones that are “present” in at least half of the samples in the enriched group using Affymetrix' MAS5 Presence/Absence (P/A) calls. We used a paired t-test when comparing samples in groups N_1_ and N_2_.

**Data deposition:** All data sets have been deposited in the Gene Expression Omnibus, www.ncbi.nlm.nih.gov/geo (accession nos. GSE10041 and GSM253663-253734).

### Gene Ontology and Gene Set Enrichment Analyses

Differentially expressed genes between the 3 groups (N_2_ vs. N_1_, M vs. N_1_ and M vs. N_2_) were separately analyzed using EASE to identify biologically relevant categories that are over-represented in the input set [21]. EASE analyses tested each list against all genes on the chip and overrepresentation describes a group of genes belonging to a certain GO category that appear more often in the given input list than expected to occur if the distribution were random. GO categories that had EASE scores of 0.05 or lower were selected as significantly over-represented. Gene Set Enrichment Analysis (GSEA 2.0 package http://www.broad.mit.edu/gsea/) was used to determine whether an *a priori* defined set of genes showed statistically significant, concordant differences between 2 groups (N_2_ vs. N_1_, and M vs. N_1_) in the context of known biological pathways. We tested expression values of all the genes in the relevant sample groups against 1687 gene sets obtained from the MSigDB2.0 for enrichment belonging to various metabolic pathways, chromosomal locations and functional sets (gene sets related to cancer/cancer cells are not included). The enriched gene sets have nominal p-value (NPV) less than 1% and False Discovery Rate (FDR) <50% after 100 random permutations. These criteria ensure that there is minimal chance of identifying false positives.

**Supplementary Methods** are located at http://bidmcgenomics.org/MIND_BODY_RR/(Login: benson) (Password: test1).
